# Sleep quality and cognitive functioning among Chinese older adults living in the US: A mixed-effects model analysis

**DOI:** 10.21203/rs.3.rs-4293848/v1

**Published:** 2024-04-29

**Authors:** Fengyan Tang, Yuyang Zhu, Dasuni Jayawardena, Guoping Jin, yanping jiang

**Affiliations:** University of Pittsburgh; The State University of New Jersey; The State University of New Jersey; University of Pittsburgh; The State University of New Jersey

**Keywords:** sleep health, cognition, older Chinese Americans, mixed-effects models

## Abstract

**Background:**

Racial and ethnic disparities in sleep quality and cognitive health are increasingly recognized, yet little is understood about their associations among Chinese older adults living in the United States. This study aims to examine the relationships between sleep parameters and cognitive functioning in this population, utilizing data from the Population Study of Chinese Elderly in Chicago (PINE).

**Methods:**

This observational study utilized a two-wave panel design as part of the PINE, including 2,228 participants aged 65 years or older, self-identified as Chinese, who completed interviews at two time points. Cognitive functioning was assessed using a battery of tests on perceptual speed, episodic memory, working memory, and mental status. Sleep quality was assessed using Pittsburgh sleep quality index (PSQI) with four aspects: subjective sleep quality, sleep latency, sleep efficiency, and sleep duration at night. Insomnia was assessed using four items from the Women’s Health Initiative Insomnia Rating Scale. Mixed-effects regression models were estimated to assess the predictive effects of sleep parameters on baseline cognitive functioning and the rate of cognitive change over time.

**Results:**

Significant negative associations were observed between poor sleep quality and baseline cognitive functioning across various domains, although these initial negative associations diminished over time. More insomnia problems were related to poorer perceptual speed and episodic memory. Long sleep latency, or a long time to sleep onset, was associated with worse functioning across all domains except mental status. Sleep efficiency showed inconsistent associations with various cognitive domains, while sleep duration showed no significant relation to any domains.

**Conclusions:**

These findings suggest that poor sleep quality indicators serve as early markers of cognitive impairments. Hence, targeted interventions aimed at improving sleep quality could potentially enhance cognitive health outcomes.

## Background

Sleep problems and cognitive decline are prevalent among older adults. Although it is well established that sleep plays a crucial role in cognitive functioning and memory consolidation [1], it remains unclear whether sleep problems predict poor cognitive functioning in later life [2]. Health disparities based on race and ethnicity may further complicate the relationship between sleep and cognition [3]. There is increasing evidence of racial/ethnic disparities in sleep quality and cognitive health [3–5], yet little is known about their associations among older Chinese Americans, a rapidly growing population.

As individuals age, their sleep patterns often undergo changes, such as reduced total sleep duration and efficiency, increased sleep fragmentation, difficulty falling asleep, decreased time spent in rapid eye movement sleep and in slow wave sleep [6]. It is estimated that up to 50% of older adults frequently experience difficulty initiating or maintaining sleep, and about 40–70% report chronic sleep problems [7–9]. These sleep problems have significant health implications in old age, including poor self-reported health, depression, cognitive decline, limitations in daily activities, reduced quality of life, and heightened risk of institutionalization [7].

Emerging evidence suggests the connections between self-reported sleep problems and cognitive functioning in older adults living in the community [10–12]. Yet, among healthy older adults, the associations between sleep problems and cognitive functioning are not always apparent [12]. Some studies yielding null results may be attributed to the misalignment of cognitive tests, imprecise measures of cognitive functioning, or inadequate assessment and analysis of sleep patterns [12]. Self-reported sleep measures include sleep latency (time to sleep onset), wake after sleep onset, sleep efficiency (ratio of hours slept to hours spent in bed), sleep duration, and general sleep complaints [10]. Cognition tests typically assess executive function, attention, episodic memory, working memory, and processing speed, collectively indicating overall cognitive functioning [10]. Multiple measures of sleep may have differential associations with cognitive functioning and its various domains. Previous studies have documented that long sleep latency and duration, poor sleep efficiency and quality, and excessive daytime sleepiness were associated with cognitive impairment in later life; however, findings on these associations have been inconsistent [13]. Therefore, further research, especially with longitudinal designs, is essential to clarify the associations between specific aspects of sleep quality and various cognitive domains.

As poor sleep health is not an integral part of the aging process, its impact varies among adults and can significantly affect cognitive health disparities, particularly among racially and ethnically marginalized groups [3, 14]. This may be especially pertinent for older Chinese Americans, who often struggle with acculturation stress due to language barriers, social isolation, and limited access to health services [15, 16]. Experiencing acculturative stress and psychological distress can contribute to sleep disturbances among older Asian and Latino Americans [3, 14, 17]. A few studies indicate that older Chinese Americans tend to report poorer sleep quality, including shorter sleep duration, lower sleep quality, and longer sleep onset, when compared to their White counterparts [16, 17].

Several studies focusing on Asian Americans have revealed the heterogeneity in the relationships between sleep and cognitive functioning across racial and ethnic groups. However, the nuanced associations between sleep problems and cognitive health warrant further investigation within each ethnic group [3]. To our knowledge, no study has specifically addressed older Chinese Americans, despite their considerable population growth and unique health disparities. Given the limited research on this population and the inconsistent findings in the broader older populations, it is important to improve our understanding of the relationship between sleep problems and cognitive functioning among older Chinese Americans to reduce health disparities. Therefore, the present study aims to investigate the associations between sleep parameters and various domains of cognitive functioning among Chinese older adults, using data from the Population Study of Chinese Elderly in Chicago (PINE). We hypothesize that poorer sleep quality and more insomnia are associated with lower levels of baseline cognition and faster rates of cognitive decline over time.

## Methods

### Study design and sample

This observational study utilized a two-wave panel design as part of the PINE, a population-based, epidemiological study aimed to examine sociocultural determinants of health among older Chinese Americans in the greater Chicago area. Details of the study design, participant recruitment, and data collection procedures have been previously published [18]. This analysis included 2,228 participants aged 65 years or older, self-identified as Chinese, who completed the interview between 2017 and 2019 (baseline or T1) and underwent follow-up between 2019 and 2021 (T2). At T1, the PINE initiated the collection of sleep measures.

The PINE study was approved by the Institutional Review Board at Rush University Medical Center in Chicago, Illinois (IRB#: 10090203). Written consent was obtained from all participants. This secondary analysis of the PINE study was approved by Institutional Review Boards at the University of Pittsburgh (IRB#: EXT20030031) and Rutgers, The State University of New Jersey (IRB#: Pro2018001578).

### Measurement

#### Cognitive functioning.

A battery of cognitive tests was conducted to gauge participants’ cognitive functioning and various domains. *Perceptual speed* was assessed using the oral version of the 11-item Symbol Digit Modalities Test (SDMT), which calls for rapid perceptual comparisons of numbers and symbols during the 90-second duration of the test. *Episodic memory* was assessed using the summary score of two tests: the East Boston Memory Test-Immediate Recall (EBMT), and the East Boston Memory Test-Delayed Recall (EBDR) of brief stories. *Working memory* was assessed using the Digit Span Backwards Test (DB), which was drawn from the Wechsler Memory Scale-Revised Test. *General mental status* was measured through the 30-item Chinese Mini-Mental State Examination (C-MMSE), which is based on the MMSE and has been widely used in epidemiological studies [19]. Based on these tests, a *global cognition* score (Cronbach’s α = 0.87) was calculated by averaging standardized scores of above tests to minimize floor and ceiling artifacts and other measurement errors [20].

#### Sleep parameters.

Sleep parameters included sleep quality with four components and insomnia. *Overall sleep quality* was assessed with the Pittsburgh Sleep Quality Index (PSQI), with a composite score comprising four components: subjective sleep quality, sleep latency, sleep efficiency, and sleep duration [21]. The PSQI is a validated and extensively adopted instrument to evaluate subjective sleep quality among older adults [22–25]. *Subjective sleep quality* was assessed with one item, i.e., during the past month, how would you rate your sleep quality overall? Responses were given on a 4-point scale, ranging from 0 (very good) to 3 (very bad). *Sleep latency* was measured with one question asking about the time it takes to fall asleep. Responses were categorized into 0 ( < = 15 mins), 1 (> 15 to = < 30), 2 (> 30 to = < 60), or 3 (> 60 mins). Based on responses to four questions, *sleep efficiency* was calculated as the actual hours of sleep time divided by the total hours in bed and multiplied by 100. It was recoded as 0 ( > = 85%), 1 ( > = 75% to < 85%), 2 ( > = 65% to < 75%), or 3 (< 65%). *Sleep duration* was measured by the actual hours slept at night, which was further recoded on as 0 ( > = 7 hours), 1 (< 7 to > = 6), 2 (< 6 to > = 5), or 3 (< 5 hours). The component scores were summed to form a global PSQI score ranging from 0 to 12, with a higher score indicating poorer sleep quality (Cronbach’s α = 0.75).

*Insomnia* was measured by four items adapted from the Women’s Health Initiative Insomnia Rating Scale (WHIIRS). WHIIRS is a reliable and valid tool to measure perceived insomnia severity in older adults [26, 27]. Participants were asked, over the past month, how often they had difficulty falling asleep, woke up at night and unable to get back to sleep, woke up too early in the morning and unable to get back to sleep, and felt excessively sleepy during the day. Each of these items were scaled on a 5-point scale ranging from 0 (never) to 4 (almost always). A sum score (range from 0 to 16) was used, with higher scores indicating more severe insomnia (Cronbach’s α = 0.83). In addition, the item about *daytime sleepiness* was taken out to be controlled in the models with the PSQI and its subdomains.

### Covariates.

Time was indicated by baseline (0) and follow-up (1). Sociodemographic covariates included age, sex, married status, education, and annual income. Immigration-related variables included years living in the U.S. and acculturation. Acculturation was measured with a 12-item multidimensional scale of individual preferred language use in different settings and preferred ethnicity they interacted with. Responses were given on a 5-point scale, from 1 (only Chinese) to 5 (only English). The summary score ranged from 12 to 60, with higher scores indicating a higher level of acculturation (Cronbach’s *α* = 0.92).

We also controlled for health behaviors and health-related variables that were thought to relate to cognitive functioning. Health behaviors included alcohol use and physical activity. Alcohol use was calculated as the average amount of alcoholic beverage consumption per day. Physical activity was assessed with the Basic Physical Activities (NAGI) Scale, which indicates difficult level of performing various physical activities (Cronbach’s α = 0.80). A higher score indicated lower level of physical activity. Health-related variables included BMI, which was categorized as normal (< 25), overweight ( > = 25 to < 30), and obese ( > = 30). Depressive symptoms were assessed using the Patient Health Questionnaire, excluding the question regarding respondents’ sleep disturbance experience (Cronbach’s α = 0.78). Instrumental Daily living limitations (IADL) were assessed with a scale measuring the difficulty in performing various instrumental activities of daily living (Cronbach’s α = 0.90).

### Statistical analysis

Mixed-effects regression models were estimated to assess initial status and changes in cognitive functioning and the predictive effects of sleep parameters after adjusting for sociodemographic variables, health behaviors, and health-related covariates. The models used sleep measures and covariates from T1, and cognitive measures from both time points. Fixed effects were used to determine whether the average change in the outcome variable was associated with a one-unit change in a predictor variable [28]. Random effects represent the general variability among subjects [28]. The effect of time was entered as a fixed factor to capture potential differences in cognitive functioning between the two time points. Sleep parameters, covariates, and their interactions with time were entered as fixed effects to assess their associations with initial level of cognition and the rate of change. Given that two data points might not adequately illustrate a change trend, random intercept models were specified to allow for individual-specific means varying around the sample mean intercept [28]. For each cognitive outcome, six mixed-effects regression models were estimated, with one sleep parameter and its interaction with time being entered respectively. All continuous variables were mean-centered to prevent multicollinearity. The analyses were performed using Stata 18.0 (29).

## Results

Table 1 presents the characteristics of the study participants. The mean age of participants was 77 years (*SD* = 7.6), with 60% being female. On average, participants had resided in the US for 25 years (*SD* = 12.0), with a mean acculturation score of 14.6 (*SD* = 3.8). Their reported average PSQI score was 4.1 (*SD* = 3.1), and the mean insomnia score was 5.5 (*SD* = 4.2). Over 60% of participants reported either good or very good sleep quality. They reported relatively few depressive symptoms (*M* = 1.5, *SD* = 2.8), and had an average IADL score of 6.4 (*SD* = 7.8).

Table 2 presents the results of six mixed-effects regression models of global cognition. The estimates of time and covariate effects reported here were derived from the model using PSQI to predict both baseline and the rate of change in global cognition (Model 1). Four out of six full models did not reveal any significant change in global cognition over the 2-year observation period, except in the models using a single item of subjective sleep quality and sleep duration (see Additional File 1). Overall, significant negative associations were observed between sleep parameters and global cognition cross-sectionally. Poorer sleep quality, as indicated by the PSQI (Model 1: *B* = −0.01, *SE* = 0.01, *p* < .01), and more insomnia symptoms (Model 2: *B* = −0.01, *SE* = 0.00, *p* < .001) were associated with lower baseline cognitive functioning, respectively. Among the subdomains in the PSQI, all except sleep efficiency were significantly related to baseline cognition. Specifically, respondents reporting very bad sleep quality exhibited worse global cognition scores compared to those reporting very good sleep quality (Model 3: *B* = −0.28, *SE* = 0.06, *p* < .001). Similarly, those taking longer to fall asleep (30 to 60 minutes) showed lower global cognition scores compared to those falling asleep within 15 minutes (Model 4: *B* = −0.13, *SE* = 0.04, *p* < .01). Those sleeping less than five hours per night demonstrated lower global cognition scores (Model 6: *B* = −0.09, *SE* = 0.04, *p* < .05), while those sleeping between 6–7 hours had higher scores (Model 6: *B* = 0.07, *SE* = 0.03, *p* < .05) when compared to those sleeping over seven hours.

Contrary to our hypothesis, sleep parameters were not associated with cognitive decline over time. Rather, we found positive time interactions with PSQI, insomnia, and subjective sleep quality. That is, these sleep measures were associated with slower rates of decline in global cognition, indicating a lessened impact of sleep problems on cognitive functioning as time progressed. Among the covariates, older age, lower levels of education, acculturation, and physical activity, as well as more IADL, were associated with worse cognitive functioning at baseline. Additionally, significant associations were observed between age and education with cognitive decline, indicating that older age and more years of education accelerated cognitive decline. Interestingly, overweighted respondents exhibited better functioning compared to those with normal weight at baseline. Daytime sleepiness showed inconsistent effects: respondents reporting sometimes feeling drowsy during the day had worse cognitive performance compared to those reporting never feeling drowsy, while those reporting always feeling drowsy exhibited better cognitive performance.

Table 3 presents the mixed-effects model estimates of sleep measures in relation to four cognitive domains after controlling for covariates. Similar to the results for global cognition, significant negative relationships were found cross-sectionally between sleep parameters and cognitive measures. Specifically, poorer sleep quality, either measured by PSQI or the single item, was associated with worse status in most domains. More insomnia problems were related to poorer perceptual speed and episodic memory. Long sleep latency was associated with worse functioning in all domains except general mental status (C_MMSE). Sleep efficiency showed inconsistent associations with various domains, while sleep duration was not significantly related to any domains. Over time, the negative effects of sleep quality may diminish in certain domains, particularly working memory and general mental status. The effects of covariates remained consistent across models (detailed results available upon request).

## Discussion

The current study examined the associations between sleep parameters and cognitive functioning in the largest population-based epidemiological study of U.S. Chinese older adults. Consistent with some previous studies [10], our findings generally supported cross-sectional negative relationships between self-reported sleep parameters and the domains of cognition and global cognition among older Chinese Americans. Self-reported sleep quality, whether assessed through the PSQI scale or one of its individual items, demonstrated significant associations with cognitive measures.

Specifically, individuals reporting very bad sleep quality demonstrated lower scores on working memory, episodic memory, and general mental status, although no difference in perceptual speed was observed between them and those reporting very good sleep quality. This is in line with a previous study that found no difference in information-processing speed between poor and good sleepers among community-dwelling, healthy older Americans [30]. These findings suggest that the neurological predictors of frontal and cerebellar gray matter are likely contributors to the age-related decline in processing speed [31].

Our study revealed distinct associations between various aspects of sleep quality and cognitive domains. Long sleep latency, indicating more difficulty initiating sleep, was significantly associated with worse global cognition, perceptual speed, episodic memory, and working memory. On the other hand, sleep duration was only linked to global cognition, while sleep efficiency was solely related to perceptual speed. The absence of significant effects of sleep efficiency on other domains of cognitive functioning may be attributed to greater measurement error associated with this variable [32, 33]. Consistent with previous research indicating that sleep durations shorter or longer than 6–7 hours were associated with worse cognition [8], we found that sleeping for 6–7 hours may be optimal for global cognition, although not necessarily for specific domains. This observation could be explained by the notion that the most critical aspect of sleep quality may be the amount of unwanted intruding wakefulness experienced, rather than the total duration of sleep [30].

We did not find significant time effect of sleep parameters on cognitive decline, with some findings even opposite to our hypothesis. Specifically, the negative associations of poor sleep quality with global cognition and working memory seemed to diminish over time. This could be attributed to the relatively short 2-year observation period, which might not have been sufficient to capture a declining trend. In our study, only general mental status consistently exhibited decline across analysis models. Further, it is plausible that chronic sleep problems rather than short-term fluctuations are linked to cognitive decline. Alternatively, the association between sleep and cognition may weaken as individuals age, as the aging brain may become less efficient in supporting sleep-specific cognitive processes [12]. If older adults experience chronic sleep disturbances or deprivation, depriving them of additional sleep might have minimal effects [12]. This could explain our findings that insomnia, characterized by chronic sleep problems, was not associated with cognitive decline, as well as the contradictory effects observed for low sleep efficiency and daytime sleepiness. These contradictory findings could potentially be attributed to individual differences in cognitive status. For some older adults, cognitive capacity may necessitate reflecting on sleep amount and quality over the past month. Poor sleep quality, including long sleep latency and low sleep efficiency, may not necessarily act as risk factors for cognitive decline, but rather serve as early markers of neurodegeneration in nondemented individuals [13]. It is also plausible that some older adults may exhibit more resistance to the cognitive effects of sleep problems, possibly due to physiological adaptations throughout the aging process [10].

There are several limitations that should be noted in the study. First, this analysis was conducted among older adults residing in the community. This means that some participants might remain relatively healthy and function well, while those with severe cognitive and physical deficits might not have been able to participate in the PINE study. Thus, health selection bias could potentially lead to underestimated negative effects of sleep problems. Second, the PINE study solely relied on self-reported measures for sleep problems. While subjective measures of sleep have been less consistently linked to poorer cognitive functioning [12], they can also introduce differential misclassification and selective drop-out. This is because individuals with poor cognitive functioning may have more difficulty accurately completing sleep questionnaires [10]. It is important to note that the PSQI, utilized as a measure of sleep quality, may not be the most suitable for older adults. Its reliance on cognitive capacity to reflect on the past month could introduce bias, especially considering the potential cognitive decline in the long run [34]. Lastly, the average 2-year follow-up period in our study was relatively short. This short duration might have limited our ability to detect cognitive decline and associated factors related to sleep problems. Future research should aim to overcome these limitations by employing longitudinal research designs and integrating reliable and valid objective measures of sleep and cognition. This will enable a more thorough understanding of changes in sleep patterns and cognitive functioning, and the factors influencing the association between sleep and cognition over time.

## Conclusion

The current study examined multiple facets of subjective sleep measures in relation to global cognition and specific cognitive domains. Our findings underscored the significant associations between sleep quality, as measured by PSQI and a single self-reported item, and various aspects of cognitive functioning. Specifically, long sleep onset latency appeared to be linked to potential impairment in episodic memory, perceptual speed, and global cognition. Given the high prevalence of sleep disturbances and cognitive disorders among older adults, understanding the relationship between sleep and cognitive aging holds significant public health implications. Targeting individuals at risk of cognitive decline through interventions aimed at improving sleep quality could potentially enhance cognitive health outcomes. Moreover, considering the racial and ethnic disparities in sleep and cognitive health, coupled with the limited research among older Chinese Americans, there is a pressing need for longitudinal and interventional studies to mitigate health inequities within this minority population. Older Chinese Americans may particularly benefit from regular health screenings, targeted sleep interventions, and engagement in various activity programs. These interventions should be implemented using a culturally sensitive, community-based approach to ensure effectiveness and accessibility for this population. By addressing sleep-related factors in cognitive health promotion efforts, we can work towards reducing disparities and enhancing overall well-being among older adults, particularly within minority communities.

## Figures and Tables

**Table 1.** Characteristics of participants at T1 (*N* = 2,228)

Characteristics	Mean (SD)/n(%)^a^	Min	Max
Global cognition	−0.37 (0.95)	−3.12	1.63
Perceptual speed	−0.42 (1.03)	−2.36	3.16
Episodic memory	−0.36 (1.09)	−2.85	1.29
Working memory	−0.20 (0.95)	−2.15	2.94
C_MMSE	−0.50 (1.37)	−5.72	1.02
Overall sleep quality - PSQI	4.13 (3.05)	0	12
Subjective sleep quality		1	4
Very good	226 (11.94)		
Fairly good	1,101 (49.42)		
Fairly bad	673 (30.21)		
Very bad	188 (8.44)		
Sleep latency		1	4
<=15 mins	875 (39.27)		
<15 to =< 30 mins	691 (31.01)		
>30 to =<60 mins	405 (18.18)		
>60 mins	257 (11.54)		
Sleep efficiency		1	4
>=85%	1,347 (60.76)		
>=75% to <85%	346 (15.61)		
>=65% to <75%	212 (9.56)		
<65%	312 (14.07)		
Sleep duration		1	4
>=7h	1,058 (47.64)		
< 7 to >= 6h	489 (22.02)		
<6 to >=5h	354 (15.94)		
<5h	320 (14.41)		
Insomnia	5.50 (4.24)	0	16
Daytime sleepiness		1	5
Never	1,064 (47.78)		
Rarely	299 (13.43)		
Sometimes	468 (21.01)		
Often	253 (11.36)		
Almost always	143 (6.42)		
Age	77.42 (7.57)	65	103
Female	1,331 (59.74)	0	1
Married status	1,437 (64.50)	0	1
Education	8.84 (5.00)	0	26
Income	2.03 (1.08)	1	10
Years living in the US	24.54 (12.01)	5.71	95.67
Acculturation	14.57 (3.83)	12	54
Alcohol use	0.02 (0.07)	0	1.02
Physical activity	5.95 (5.60)	0	20
BMI^a^			
Normal	1,585 (71.36)	1	3
Overweight	553 (24.90)		
Obese	83 (3.74)		
Depression	1.50 (2.78)	0	22
IADL	6.42 (7.85)	0	36

Note.

a Frequencies and percentages were reported for categorical variables.

**Table 2.** Fixed Effects of Associations between Sleep Parameters and Global Cognition

Predictors	Coefficient (SE)	*p*
***Model 1***		
Time	−0.02 (0.02)	0.28
Age	−0.02 (0.00)	<0.001
Age*time	−0.00 (0.00)	<0.01
Female	0.05 (0.03)	0.12
Female*time	−0.02 (0.01)	0.15
Married	0.05 (0.03)	0.11
Married*time	0.02 (0.02)	0.13
Education	0.08 (0.00)	<0.001
Education*time	−0.00 (0.00)	<0.05
Income	0.01 (0.01)	0.54
Income*time	−0.00 (0.01)	0.83
Acculturation	0.02 (0.00)	<0.001
Acculturation*time	0.00 (0.00)	0.61
Years in the US	0.00 (0.00)	0.60
Years in the US*time	0.00 (0.00)	0.23
Physical activity	−0.03 (0.00)	<0.001
Physical activity*time	0.00 (0.00)	0.52
Alcohol use	−0.03 (0.20)	0.90
Alcohol use*time	0.09 (0.10)	0.37
BMI		
Normal (ref)		
Overweight	0.08 (0.03)	<0.01
Obese	−0.03 (0.07)	0.65
BMI*time		
Normal (ref)		
Overweight	−0.00 (0.02)	0.97
Obese	0.04 (0.03)	0.28
Depression	−0.01 (0.01)	0.09
Depression*time	0.00 (0.00)	0.12
IADL	−0.03 (0.00)	<0.001
IADL*time	0.00 (0.00)	0.66
Daytime sleepiness^a^		
Never (ref)		
Rarely	−0.04 (0.04)	0.34
Sometimes	−0.09 (0.04)	<0.05
Often	−0.08 (0.05)	0.08
Almost always	0.13 (0.06)	<0.05
Daytime sleepiness*time^a^		
Never (ref)		
Rarely	0.01 (0.02)	0.75
Sometimes	−0.01 (0.02)	0.55
Often	0.04 (0.02)	0.09
Almost always	−0.03 (0.03)	0.27
PSQI	−0.01 (0.01)	<0.01
PSQI*time	0.01 (0.00)	<0.05
***Model 2***		
Insomnia	−0.01 (0.00)	<0.001
Insomnia*time	0.00 (0.00)	<0.05
***Model 3***		
Subjective sleep quality		
Very good (ref)		
Fairly good	−0.04 (0.04)	0.35
Fairly bad	−0.04 (0.05)	0.42
Very bad	−0.28 (0.06)	<0.001
Subjective sleep quality*time		
Very good (ref)		
Fairly Good	0.06 (0.02)	<0.01
Fairly Bad	0.05 (0.02)	<0.05
Very Bad	0.09 (0.03)	<0.01
***Model 4***		
Sleep latency		
<=15 min (ref)		
15–30 min	0.05 (0.03)	0.09
30–60 min	−0.13 (0.04)	<0.01
>60 min	−0.08 (0.05)	0.09
Sleep latency*time		
<=15 min (ref)		
15–30 min	0.01 (0.02)	0.74
30–60 min	0.02 (0.02)	0.31
>60 min	−0.00 (0.02)	0.97
***Model 5***		
Sleep efficiency		
>=85% (ref)		
75–85%	0.05 (0.04)	0.16
65–75%	0.05 (0.05)	0.32
<65%	−0.08 (0.04)	0.06
Sleep efficiency*time		
>=85% (ref)		
75–85%	0.00 (0.02)	0.99
65–75%	0.01 (0.02)	0.60
< 65%	0.02 (0.02)	0.30
***Model 6***		
Sleep duration		
>=7h (ref)		
6–7h	0.07 (0.03)	<0.05
5–6h	−0.02 (0.04)	0.62
<5h	−0.09 (0.04)	<0.05
Sleep duration*time		
>=7h (ref)		
6–7h	0.03 (0.02)	0.07
5–6h	0.04 (0.02)	<0.05
<5h	0.04 (0.02)	0.07

*Note.* Model 2 controlled for all covariates except daytime sleepiness and the interaction between daytime sleepiness and time, considering daytime sleepiness as one item measuring insomnia. Models 3 to 6 controlled for all covariates as those in Model 1.

**Table 3.** Fix Effects of Associations between Sleep Parameters and Cognitive Domains.

	Perceptual speed	Working memory	Episodic memory	C_MMSE
Predictors	Coefficient (SE)	Coefficient (SE)	Coefficient (SE)	Coefficient (SE)
**Models: PSQI + Covariates**				
Time	−0.04 (0.02)	−0.02 (0.02)	0.02 (0.03)	−0.07 (0.03)*
PSQI	−0.01 (0.01)*	−0.02 (0.01)**	−0.02 (0.01)*	−0.01 (0.01)
PSQI*time	0.00 (0.00)	0.01 (0.00)*	0.00 (0.00)	0.01 (0.00)*
**Models: Insomnia + Covariates**				
Time	−0.03 (0.02)	−0.03 (0.02)	0.03 (0.03)	−0.09 (0.03)**
Insomnia	−0.02 (0.00)***	−0.01 (0.00)	−0.02 (0.00)***	0.00 (0.01)
Insomnia*time	0.00 (0.00)*	0.00 (0.00)	0.00 (0.00)	0.00 (0.00)
**Models: Subjective sleep quality + Covariates**				
Time	−0.05 (0.03)	−0.14 (0.03)***	−0.02 (0.04)	−0.14 (0.04)**
Sleep quality				
Very good (ref)				
Fairly good	0.03 (0.05)	−0.12 (0.05)*	−0.06 (0.06)	0.00 (0.07)
Fairly bad	0.03 (0.06)	−0.14 (0.06)*	−0.04 (0.07)	−0.03 (0.07)
Very bad	−0.08 (0.08)	−0.24 (0.08)**	−0.37 (0.09)***	−0.32 (0.10)**
Sleep quality*time				
Very good (ref)				
Fairly Good	0.01 (0.02)	0.12 (0.03)***	0.05 (0.03)	0.06 (0.03)
Fairly bad	0.00 (0.03)	0.13 (0.03)***	0.04 (0.04)	0.06 (0.04)
Very bad	0.02 (0.04)	0.16 (0.04)***	0.08 (0.05)	0.10 (0.05)*
**Models: Sleep latency + Covariates**				
Time	−0.04 (0.02)	−0.04 (0.03)	0.02 (0.03)	−0.09 (0.03)**
Sleep latency				
<=15min (ref)				
15–30 min	0.01 (0.04)	0.05 (0.04)	0.07 (0.04)	0.06 (0.05)
30–60 min	−0.16 (0.05)***	−0.08 (0.05)	−0.16 (0.05)**	−0.09 (0.06)
>60min	−0.12 (0.05)*	−0.13 (0.06)*	−0.07 (0.06)	0.01 (0.07)
Sleep latency*time				
<=15min (ref)				
15–30 min	0.00 (0.02)	0.01 (0.02)	0.00 (0.02)	0.01 (0.03)
30–60 min	0.01 (0.02)	0.02 (0.03)	0.01 (0.03)	0.03 (0.03)
>60 min	−0.05 (0.03)	0.04 (0.03)	−0.02 (0.04)	0.01 (0.04)
**Models: Sleep efficiency + Covariates**				
Time	−0.05 (0.02)*	−0.04 (0.03)	0.02 (0.03)	−0.09 (0.03)**
Sleep efficiency				
>=85% (ref)				
75–85%	−0.02 (0.04)	0.01 (0.05)	0.10 (0.05)*	0.07 (0.06)
65–75%	0.07 (0.05)	−0.04 (0.06)	0.04 (0.06)	0.14 (0.07)*
<65%	−0.11 (0.05)*	−0.05 (0.05)	−0.07 (0.06)	−0.10 (0.06)
Sleep efficiency*time				
>=85% (ref)				
75–85%	0.00 (0.02)	0.01 (0.02)	−0.02 (0.03)	0.02 (0.03)
65–75%	−0.02 (0.03)	0.03 (0.03)	0.01 (0.04)	0.04 (0.04)
< 65%	0.05 (0.02)*	0.01 (0.03)	0.01 (0.03)	0.04 (0.03)
**Models: Sleep duration + Covariates**				
Time	−0.05 (0.02)*	−0.04 (0.03)	0.01 (0.03)	−0.10 (0.03)**
Sleep duration				
>=7h (ref)				
6–7h	0.04 (0.04)	0.06 (0.04)	0.09 (0.05)	0.07 (0.05)
5–6h	−0.02 (0.05)	−0.04 (0.05)	−0.01 (0.05)	−0.04 (0.06)
<5h	−0.08 (0.05)	−0.10 (0.05)	−0.10 (0.06)	−0.06 (0.07)
Sleep duration*time				
>=7h (ref)				
6–7h	0.03 (0.02)	0.03 (0.02)	0.03 (0.03)	0.06 (0.03)*
5–6h	0.01 (0.02)	0.04 (0.03)	0.04 (0.03)	0.07 (0.03)*
<5h	0.03 (0.03)	0.03 (0.03)	0.05 (0.03)	0.05 (0.03)

*Note.* All models controlled for every covariate, except for excluding daytime sleepiness from the models that utilized insomnia to predict cognitive outcomes.

* p < .05

** p < .01

*** p < .001.

## Data Availability

The data are available upon request to Dr. Yanping Jiang (yanping.jiang@ifh.rutgers.edu) Restrictions may apply.

## Abbreviations

PINE: Population Study of Chinese Elderly in Chicago
C_MMSE: Chinese Mini-Mental State Examination
PSQI: Pittsburgh Sleep Quality Index
BMI: body mass index
IADL: Instrumental Activities of Daily Living
